# Paediatric motor difficulties and internalising problems: an integrative review on the environmental stress hypothesis

**DOI:** 10.3389/fped.2024.1320338

**Published:** 2024-08-02

**Authors:** Noah Erskine, Jaime Barratt, John Cairney

**Affiliations:** ^1^ School of Human Movement and Nutrition Sciences, University of Queensland, St Lucia, QLD, Australia; ^2^ Health and Well-Being Centre for Research Innovation, University of Queensland, St Lucia, QLD, Australia; ^3^ Faculty of Education, Brock University, St. Catharines, ON, Canada

**Keywords:** motor coordination, mental health, developmental coordination disorder, paediatric comorbidities, obesity, peer problems, neurodevelopmental disorders

## Abstract

The current study aims to provide an in-depth analysis and extension of the Environmental Stress Hypothesis (ESH) framework, focusing on the complex interplay between poor motor skills and internalising problems like anxiety and depression. Using an integrative research review methodology, this study synthesises findings from 38 articles, both empirical and theoretical, building upon previous foundational works. The hypothesis posits that poor motor skills serve as a primary stressor, leading to internalising problems through various secondary stressors. A rigorous comparison of data was conducted, considering study design, findings, and methodologies—while exploring variables such as age, sex, and comorbidities. The study also enhances the ESH framework by including intrapersonal stressors and introducing resource buffers, including optimism and familial support as additional influencing factors. This multi-level approach yields a more nuanced and comprehensive ESH framework, highlighting the need for future studies to consider variables that intersect across multiple domains and how the relationship between poor motor skills and internalising problems may vary across different life stages.

## 1 Introduction

Developmental coordination disorder (DCD) is a neurodevelopmental disorder that can significantly impact the overall development of a child. Population prevalence is between 5% and 6% of all children, with 2% of children with DCD severely compromised by the disorder (1). This makes DCD one of the most prevalent childhood disorders globally (2). Although children with DCD tend to achieve basic motor milestones (e.g., sitting up and walking) at developmentally appropriate times, they have difficulty learning motor skills that require higher levels of coordination [e.g., holding and using a pencil (3)]. These children are often described by their social networks (e.g., parents & teachers) as “clumsy” or “awkward”, however, phenotypically they are defined as struggling with the mastery of skills associated with play, self-care, and academic performance. Despite having ample developmentally appropriate opportunities and experiences to learn and practice, these struggles are significant and persist throughout the lifespan for these children (2, 4, 5).

Throughout childhood, attaining developmentally suitable movement milestones plays a pivotal role in psychosocial development (6–8). Mancini et al. (9), explain that such development for both structured and unstructured play, not only galvanises fine-tuning of pre-existing motor skills and improves self-competence, but also enhances positive peer relationships through more advanced social engagement and interaction. The profoundness of such can be illustrated in classroom settings, where along with being one the first environments a child socialises and plays with a sizable number of peers, the actual execution of school-related motor skills (e.g., handwriting) also facilitates further cognitive and academic development (3, 10, 11). Consequently, children who lack developmentally appropriate motor skills struggle to fit into their environments (2, 4).

Considering the significance of developing motor skills for psychosocial development, it is unsurprising that a growing body of evidence shows a link between lower motor proficiency (i.e., poor motor skills, including DCD) and adverse psychosocial outcomes (12–15). Skinner & Piek (16), for example, illustrate that children and adolescents diagnosed with DCD reported having poor self-competence, less social support, and greater levels of anxiety in contrast to their age-matched controls. The adverse impact of poor motor skills on psychosocial functioning often persists or even intensifies over time (7, 17, 18), with studies on adults with poor motor skills showing pronounced psychosocial problems compared to typically developing peers (19–21). Among the many different psychosocial and mental health outcomes, internalising problems remain a focal interest for researchers (5, 7, 22). Internalising problems simply defined are symptoms consistent with diagnosable psychiatric conditions within the internalising spectrum, such as anxiety and depression (2, 5). Cairney et al. (22) explain that even in the absence of a clinically diagnosed internalising disorder (i.e., anxiety/depression), internalising symptoms can negatively affect overall functioning and quality of life. Piek et al. (7) and Sigurdsson et al. (23) explain that early childhood difficulties with motor skills can serve as a predictor for later internalising problems. Thus, internalising problems are frequently reported in both children and adults with motor deficits, as evidenced by numerous studies highlighting these issues across different age groups (5, 7, 11, 17, 20).

The Environmental Stress Hypothesis [ESH; (5, 22)] is the most comprehensive model in the extant literature, detailing various pathways between DCD and internalising problems. Introduced by Cairney et al. (5), the ESH postulates that motor coordination problems from DCD are primary stressors that drive a host of negative physical and psychosocial outcomes termed secondary stressors. These secondary stressors can then produce negative appraisals of self and contribute to increased internalising problems. The ESH was later refined by Cairney et al. (22) offering an explicit framework inspired by Pearlin's (24) seminal Stress Process Model.

**Figure 1:**
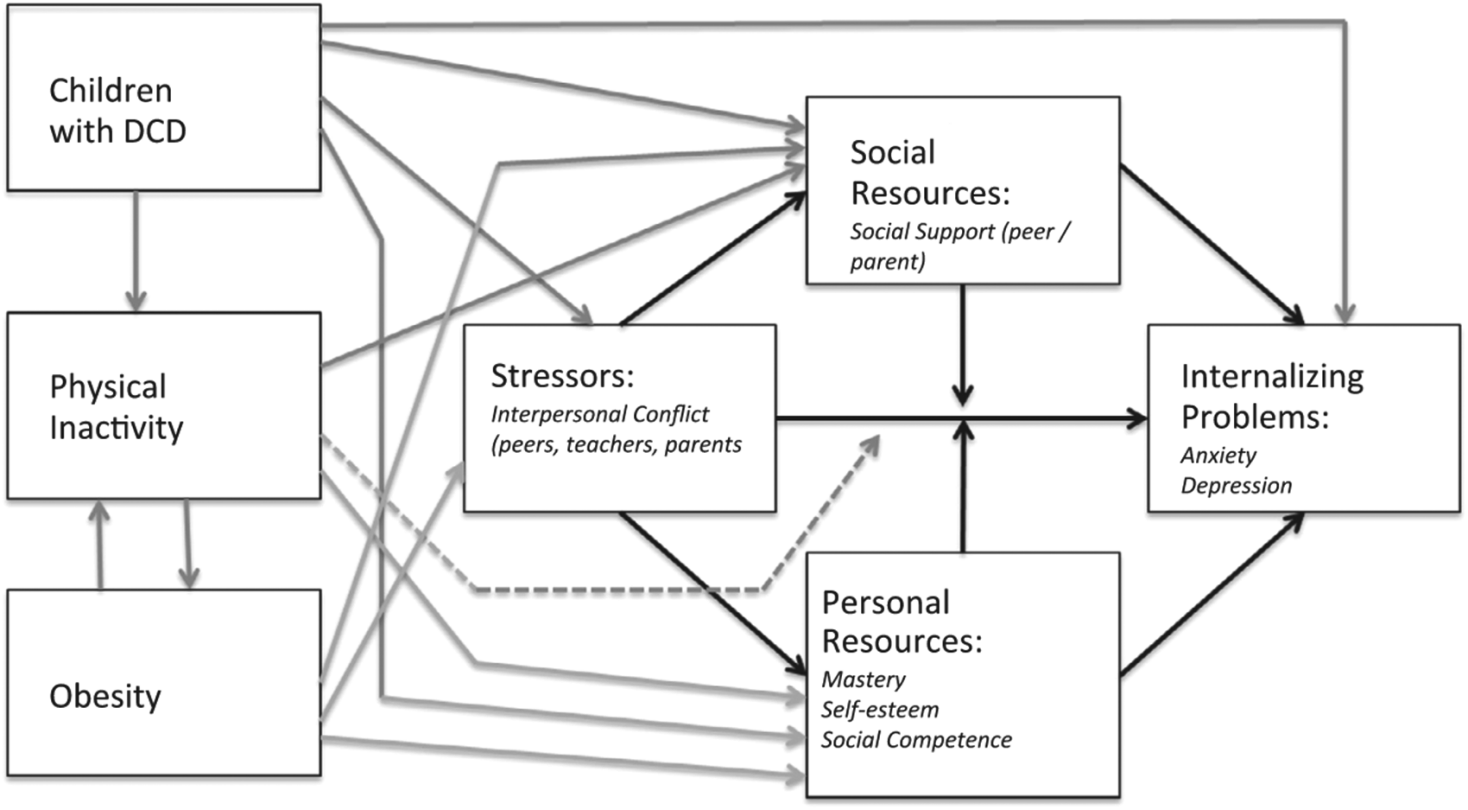
The environmental stress hypothesis (22).

The revised ESH model, depicted in Figure 1. Illustrates Pearlin's (24) original paths in the dark arrows, and Cairney et al.'s (22) additions in the grey arrows. DCD (or poor motor skills), as a primary stressor, can either elevate internalising problems or lead to secondary stressors (e.g., interpersonal conflicts) that heighten these problems. The model features various stress buffers (i.e., protective effects) in the form of social resources (e.g., social support networks) and personal resources (e.g., mastery, self-esteem and social competence) that can help dampen the effect of stressors. The ESH also considers physical inactivity and obesity which can affect intermediary pathways (e.g., social support, personal resources and interpersonal conflicts). Physical inactivity and obesity are represented with bidirectional arrows as physical inactivity can further increase obesity risk, creating a feedback loop. The broken arrow from physical inactivity to the stress pathway was added due to the association between sedentary behaviours and subsequent internalising problems. It is important to note that the ESH is a heuristic or a guiding framework for understanding the relationship between DCD and internalising problems, rather than a strict causal or mechanistic model.

The ESH framework and its proposed pathways have garnered notable empirical support [e.g., (25)]. The breadth of support is outlined in a recent empirical review by Mancini et al. (9). Despite its influence on the field, many pathways of the model remain under-examined and/or require further specification (e.g., does social support have a mediating or moderating effect?). Furthermore, previous research has shown evidence of additional factors that could potentially be added to the model such as person-level interactions in the form of age and sex differences (26, 27). Emerging evidence of novel pathways further compound the complexity of the relationship. Therefore, further research is needed to elucidate the mechanisms underlying the relationship between DCD and internalising problems. This is best accomplished by continually investigating the pathways proposed by the ESH and theoretically developing the framework. The current study builds from the review by Mancini et al. (9) investigating studies on the ESH up-to-date and by considering additional articles that were not included in the previous review. Specifically, this study aims to: (1) review the ESH literature, (2) summarise the general findings surrounding the framework to date, and (3) propose extensions to the model to better reflect the literature and guide future empirical studies.

To remain consistent with current literature and the terms used by Mancini et al. (9), the following definitions are provided: A “mediator variable” is a tertiary variable that helps explain the relationship between a predictor and an outcome variable. A “moderator variable” affects the strength and relationship between the predictor and outcome variable. “Complex connections” refer to relationships that are mediated and/or moderated by other factors, distinct from unilateral associations. A “direct relationship” refers to an association between two variables that is neither mediated nor moderated by another external variable.

## 2 Methods

### 2.1 Design

The current study used an integrative review. The aim of an integrative review is to make informed generalisations about a topic by analysing, evaluating and synthesising multiple studies on the same subject (28, 29). While a systematic review may be useful in objectively analysing and evaluating studies, the protocols for this type of review are too proscriptive, drastically limiting the ability to consider a wider range of methodologies and designs (30). Due to the complexity of the ESH, which posits the influence of multiple factors along with multiple complex pathways, we felt that the ESH framework could not be adequately investigated by reviewing a singular type of study design. Therefore, we felt that an integrative research review, which is particularly suitable for studying complex frameworks, afforded us the flexibility to consider a wider range of study designs and methodologies such as qualitative, quantitative, and mixed methods to better characterise the phenomena of concern. Moreover, the holistic nature of an integrative review further enabled us to broadly investigate the relationship between poor motor skills and psychosocial development across the full continuum of motor skills and lifespan that the relationship is explained to exist on (9, 31–34).

### 2.2 Procedure

The researchers of this study followed the steps outlined in Torraco, (35): (1) defining the research aims and problem (see above), (2) conducting a literature search and collecting data, (3) evaluating and analysing the data, and (4) presenting the findings and discussing the results.

Two reviewers were used for the scoping process of relevant literature. Search terms for the review were developed by both reviewers and with the consultation of a university librarian (see Supplementary Table S1.1 for a list of search terms).

These terms were used by the reviewers to conduct a computerised literature search of peer-reviewed studies using the databases PUBMED, COCHRANE, EMBASE, CIAHL, PsychInfo, Keele Web of Science, CDR/DARE and ProQuest. Relevant citations were then compiled from each database onto the referencing software Zotero. Once all duplicate references were removed, citations were transferred to the Covidence review software, where two reviewers (NE, JB) proceeded to independently screen on the three separate levels of titles, abstracts, and full texts based on the following inclusion criteria: (1) empirical studies testing complex relationships between variables included in the ESH or relevant works adding to or critiquing the ESH framework, (2) peer-reviewed and published (or in-press) in English language scholarly journals, and (3) published (or in-press) between 2010 and 2022. Both reviewers performed a forward-citation search of Cairney et al. (5, 22) publications in order to find supplementary papers of interest, as well as consulting leading experts in the field for other recommendations. Meta-analyses and systematic reviews, such as Mancini et al. (9), were excluded from inclusion but were used to inform the current study's protocols to make sure that the procedure remained consistent with the literature.

### 2.3 Appraisal of study quality

Study quality of all the included titles were evaluated independently by each reviewer (NE, JB) via a modified critical appraisal skill programme adapted from Mancini et al. (9) and Wilson et al. (36) (see Supplementary Table S1.2 for the modified critical appraisal).

Assessment of quality was limited to studies that had either experimental or observational research designs with quantitative measurements. All ineligible papers (i.e., reviews, critiques, qualitative assessments, and editorials) were flagged and were used as commentary surrounding the conceptualisation and utilization of the model. Eligible studies were scored out of 10, each score relating to an item rated as 1 (confirmed) or 0 (unconfirmed). The rating scale consisted of high quality (scores 8 and above), moderate quality (scores 5–7) and low quality [4 and below (9, 36)].

### 2.4 Data analysis and synthesis

To effectively organise and assess the included studies, the reviewers compared and contrasted studies, including aspects such as study design (e.g., longitudinal, cross-sectional), sample size, and other pertinent details, with the entire review team ensuring consistency and accuracy. Information on population characteristics, methodologies, and findings were meticulously recorded and detailed in Table 1. This enabled us to examine various variables such as age, disorder, and comorbidities to identify patterns and themes within the data, as reflected in the Results section.

**Table 1:** Summary of included papers (*N* = 38).

Authors, year	Use of ESH^a^	Study design	Population characteristics					Key findings
			*N*	Target group		Country	Disability	
				M age	Sex			
Blank et al. (4)	International recommendations	Editorial	N/A	N/A	N/A	International	DCD	Thirty-five recommendations were made. They cover topics such as diagnosis, assessment, intervention, psychosocial issues, and considerations for adolescents and adults with DCD. First international recommendations to consider adolescents and adults.
Bulten et al. (37)	Test of ESH	Cross-sectional (Correlational)	507	59.3 months	288 M; 219 F	Canada	DCD	Sedentary behaviour moderated the relationship between being at risk for DCD and anxiety/depression scores [*ΔR*^2^ = 0.01, F (1, 500) = 4.31, *p* = .038], particularly when sedentary behaviour was one SD above the mean.
Cacola, (38)	Reference the ESH	Literature review	N/A	N/A	N/A	N/A	N/A	DCD has mental and physical health consequences beyond motor skills. Potential mediators include peer relationships, bullying, and self-esteem. Further research is needed to understand pathways and establish causality.
Campbell et al. (39)	Commentary	Case report of participatory action research	N/A	N/A	N/A	N/A	N/A	Implementing DCD interventions in schools is challenging. The ESH model helped identify factors exacerbating poor mental health. Participatory research and knowledge translation provided insights into practical implementation and stakeholder perspectives.
Gasser-Haas et al. (40)	Test of ESH	Cross-sectional (Correlational)	Time 1: 293; Time 2: 293; Time 3: 189	Time 1 = 2.81 years; Time 2 = 3.76 years; Time 3 = 9.69 years	Time 1: 47.9% F; Time 2: 47.3% F; Time 3: 48.6% F	Switzerland	DCD	Peer problems mediated the relationship between motor performance and internalizing problems (total effect: *ß* = −0.47, *p* < 0.001; indirect effect with mediator: *ß* = −0.26, *p* = 0.13). Popularity did not mediate the effect of peer problems on internalizing problems (*β* = 0.06, *p* = 0.70). Best friendship quality had a positive moderation effect (*β* = 0.21, *p* = 0.15).
Goulardins et al. (41)	Provide evidence for ESH	Cross-sectional (Correlational)	129	11.2 years	91 M; 38 F	Australia	ADHD	Motor skills significantly predicted social problems in the teacher model (Stage 1). After controlling for ADHD symptoms, motor skills remained significant in the teacher model but not in the parent model. Inattentive symptoms were a significant predictor in the parent model, while hyperactivity/impulsive symptoms were significant in the teacher model.
Green et al. (42)	Commentary	Literature review	N/A	N/A	N/A	N/A	N/A	Explores physical and emotional coherence in DCD using the ESH model. A multi-dimensional, transactional approach considering person, activity, and environment is proposed to understand non-motor difficulties.
Harrowell et al. (43)	Test of ESH	Longitudinal (Correlational)	DCD = 168; Control = 3,750	Time 1 Range = 7 to 8 years; Time 2 Range = 16–18 years	1,889 M; 1,861 F	UK	DCD	Childhood DCD was associated with increased mental health (e.g., SDQ Peer Problems Subscale: significantly higher in the DCD group with an OR of 2.14) and social difficulties in adolescence. The relationship was partially mediated by social communication difficulties, with females at a higher risk of mental health issues compared to males.
Hill et al. (44)	Provide evidence for ESH	Cross-sectional (Correlational)	DCD = 36; Typical = 49	DCD = 29.29 (19–59); TD = 27.84 (18–56) years	DCD = 15 M, 21F; Typical = 24M, 25 F	UK	DCD; No DCD	Adults with DCD reported significantly higher levels of state anxiety [F(1,79) = 23.386, *p* < 0.001, partial *η*^2^ = 0.228], trait anxiety [F(1,79) = 43.455, *p* < 0.001, partial *η*^2^ = 0.355], and depression [F(1,79) = 25.463, *p* < 0.001, partial *η*^2^ = 0.244] compared to typical adults.
James et al. (45)	Provide evidence for ESH	Cross-sectional (Correlational)	589	4.93 years	338 M	Canada	DCD; No DCD	Children at risk for DCD showed higher internalizing (*p* < 0.001, d = 0.35) and externalizing (*p* < 0.001, d = 0.46) problems. Internalizing problems mediated the relationship between DCD risk status and fitness outcomes (significant indirect effects for internalizing problems on TTT, PP, and MP).
Li et al. (46)	Test of ESH	Cross-sectional (Correlational)	1,206	13.4 years	611 M; 595 F	Canada	DCD; No DCD	The study supported the interaction of physical activity and global self-worth in moderating the relationship between DCD and internalizing problems (significant three-way interaction of pDCD by physical activity by global self-worth, coefficient = 0.052, SE = 0.024, *p* < 0.05), with significant sex differences observed. The moderated moderation model accounted for 14.5% of the variance in internalizing problems.
Li et al. (26)	Test of ESH & Expand	Cross-sectional (Correlational)	1,206	13.4 years	612 M; 595 F	Canada	DCD; No DCD	The study supports the sequential mediation of physical activity, BMI, and global self-worth in the relationship between pDCD and internalizing problems, with notable sex differences in these pathways. The final model accounted for 21.7% of the variance in internalizing problems (e.g., χ^2^ = 2.970, df = 2, *p* = 0.227; RMSEA = 0.020).
Li et al. (47)	Test of ESH	Cross-sectional (Correlational)	225	19.5 (17–23) years	75.1% F	Canada	Neurological/ Musculoskeletal Disease and Visual Imapirment	Poor motor coordination was associated with increased psychological distress, mediated by secondary stressors and perceived social support. The ESH model modifications improved the fit (χ^2^ = 83.24, *p* < .01; RMSEA = 0.056; NNFI = 0.927; CFI = 0.954; GFI = 0.947), but extended models did not show additional improvements.
Li et al. (48)	Test of ESH	Cross-sectional (Correlational)	rDCD = 233; TD = 274	rDCD = 4.9; TD = 5 years	rDCD = 157; TD = 131	Canada	DCD; No DCD	Physical activity and BMI did not mediate the relationship between rDCD status and internalizing problems in preschool children, though rDCD status was directly associated with internalizing problems.
Mancini et al. (31)	Test of ESH	Cross-sectional (Correlational)	93	14.21 years	55 M; 38 F	Australia	Normative Sample	Significant negative correlation between motor skills and anxiety (*r* = −0.32) and depression (*r* = −.33). Perceived family support (indirect effect = −0.34, 95% CI [−0.69, −0.03) and significant other support (indirect effect = 0.19, 95% CI [0.01, 0.47) mediated the association between motor skills and depression, but not anxiety.
Mancini et al. (27)	Test of ESH	Longitudinal (Correlational)	Time 1 = 197; Time 2 = 107	Time 1 = 5.40 years; Time 2 = 6.91 years	Time 1 = 102M, 95 F; Time 2 = 57M, 50F	Australia	Normative Sample	Significant negative correlation between motor skills and internalizing problems at Time 1 (*r* = −.17) and Time 2 (*r* = −.20). Peer problems mediated this association at Time 1 (indirect effect = −0.05, 95% CI [−0.09, −0.03), while peer problems (indirect effect = −0.07, 95% CI [−0.11, −0.03) and perceived physical competence (indirect effect = −0.01, 95% CI [−0.02, −0.001) mediated this association at Time 2.
Mancini et al. (49)	Provide evidence for ESH	Cross-sectional (Correlational)	164	9.93 years	80 M; 84F	Australia	Normative Sample	Peer problems and several dimensions of perceived competence (scholastic, athletic, physical appearance, and behavioral) significantly mediated the relationship between motor skills and internalizing problems (e.g., peer problems: indirect effect = −0.02, 95% CI [−0.04, −0.002]; perceived scholastic competence: indirect effect = −0.08, 95% CI [−0.15, −0.005).
Mancini et al. (50)	Provide evidence for ESH	Cross-sectional (Correlational)	72	9.86 years	66 M	Australia	ADHD	Motor skills significantly predicted social problems in the teacher model (Stage 1). After controlling for ADHD symptoms, motor skills remained significant in the teacher model but not in the parent model. The interaction term between motor coordination and sleep difficulties was marginally non-significant (*p* = .06) in predicting peer problems.
de Medeiros et al. (51)	Provide evidence for ESH	Cross-sectional (Correlational)	431	8.97 years	191 M; 240 F	Brazil	Disabilities	Externalizing problems significantly mediated the relationship between low motor proficiency and internalizing problems (indirect effect = 0.01, bootstrap SE = 0.058, 95% CI = 0.029 to 0.009). Other potential mediators (BMI, physical activity, self-efficacy, perceived social status, prosocial behavior, HRQOL) did not show significant mediation effects (all 95% CIs for indirect effects included zero).
Missiuna et al. (25)	Provide evidence for ESH	Cross-sectional (Correlational)	244	11.9 years	146 M; 40 F	Canada	DCD; ADHD; TD	Children with DCD, ADHD, and comorbid DCD/ADHD experienced higher levels of depression (parent report, F(3,236) = 23.7, *p* < 0.001; child report, F(3,238) = 9.9, *p* < 0.001) and anxiety (parent report, F(3,235) = 8.9, *p* < 0.001; child report, F(3,236) = 5.6, *p* = 0.001) compared to typically developing children, after controlling for age, sex, and caregiver ethnicity.
Missiuna et al. (25)	Commentary & Extension	Literature review	N/A	N/A	N/A	N/A	N/A	Children with DCD face social and emotional problems. A multi-disciplinary approach extending beyond the individual is needed for assessment and intervention. Longitudinal studies should explore DCD's influence on health and weight.
Nobusako et al. (52)	Provide evidence for ESH	Experimental	61	DCD = 9.4 years Control = 9.3 years	M only	Japan	DCD; No DCD	Children with DCD had a longer PSE (time window for Sense of Agency) compared to typically developing children. PSE was positively correlated with depressive tendency in the DCD group.
Noordstar et al. (53)	Provide evidence for ESH	Experimental	31	8 years	21 M; 10F	Netherlands	DCD	Care-as-usual treatment was just as effective as the combined intervention for improving motor performance and self-perceptions in children with DCD (no significant effect of the intervention on motor performance, perceived athletic competence, global self-esteem, leisure physical activity, and total physical activity). However, perceived self-competence did not improve, and physical activity levels remained unchanged.
Omer and Leonard, (54)	Provide evidence for ESH	Cross-sectional (Correlational)	83	DCD = 12.45; Control = 11.98 years	DCD (25 M; 7 F) and Control (28 M; 23 F)	UK	DCD; No DCD	Executive function (EF) difficulties significantly mediated the relationship between DCD and internalizing symptoms (indirect effect = 14.20, BC 95% CI [7.12, 21.02), particularly through behavioral regulation difficulties.
Piek et al. (55)	Provide evidence for ESH	Experimental	Time 1 = 486; Time 2 = 456; Time 3; 337	Time 1 = 5.42 (4.83–6.17) years	257 M; 254F	Australia	Normative Sample	The Animal Fun intervention program significantly improved prosocial behavior [significant Group × Time interaction, F(21,262) = 4.34, *p* = .013] in young children but did not significantly improve internalizing problems. Improvements in prosocial behavior were more pronounced in females [significant 3-way Group × Time × Sex interaction, F(21,254) = 7.61, *p* = .001].
Rigoli et al. (20)	Test of ESH	Cross-sectional (Correlational)	95	21.73 years	35M; 60 F	Australia	Normative sample	Significant negative correlation between motor skills and internalizing problems (*r* = −.27). Perceived social support mediated the relationship (indirect effect = .06, *p* = .003), but not physical self-worth.
Rigoli et al. (56)	Provide evidence for ESH	Literature review	N/A	N/A	N/A	N/A	N/A	Motor skill difficulties may increase mental health risks, but this relationship is under-recognized. Increased awareness and screening are needed to improve assessment, intervention, and outcomes.
Rigoli et al. (57)	Provide evidence for ESH	Cross-sectional (Correlational)	93	14.21 years	55M; 38 F	Australia	Normative sample	Significant negative correlation between motor skills and Emotional Functioning (*r* = −.47). Self-perceptions mediated the association between motor skills and Emotional Functioning, with motor coordination accounting for 44% of the variance in overall psychosocial well-being.
Rodriguez et al. (58)	Provide evidence for ESH	Cross-sectional (Correlational)	582	5 years	334 M; 248 F	Canada	rDCD (At risk for DCD); No DCD	Preschool children at risk for motor delays exhibited higher levels of emotional and behavioral problems, with a higher likelihood of comorbidity, particularly for depression (significant difference in BDI categories [*χ*^2^ (3) = 25.837, *p* < 0.001] and autism.
Tal Saban & Kirby, (59)	Provide evidence for ESH	Cross-sectional (Correlational)	212	22.37 years	M 34%	UK	DCD; No DCD; ASD; ADHD	Empathy levels in adults with DCD were similar to typically developing adults [DCD group showed similar EQ scores to the control group; F (4,257) = 35.63; *p* < 0.001]. High correlations were found between EQ and both parts of the SAF-Q (SAF-Q “past” and “currently”).
Tal-Saban et al. (60)	Provide evidence for ESH	Cross-sectional (Correlational)	52	GDD & DCD = 4.92; GDD = 5.09 years	GDD & DCD∼16 M; GDD∼18 M	Israel	DCD; GDD	Children with GDD and DCD had more social skill difficulties (significant overall group effect) compared to children with GDD only. Kindergarten teachers reported more difficulties in both social skills (significant main effect of reporter) and behavioral problems (significant main effect of reporter) than parents did.
Taylor et al. (61)	Provide evidence for ESH	Cross-sectional (Correlational)	112	EBD = 10.3 years	67 M; 45 F	UK	EBD, no EBD	Children with severe Emotional and Behavioral Difficulties (EBD) had significant motor skill difficulties (significant effect of group on motor skills; MABC-2) and primary reflex persistence (significant effect of group on arm:head movement ratios; Schilder Test for ATNR persistence), which explained 70% of the variation in psychosocial functioning (hierarchical multiple regression).
Viholainen et al. (62)	Provide evidence for ESH	Cross-sectional (Correlational)	327	12–16 years	327F	Finland	Normative sample	The study highlighted the significant role of motor skills in explaining psychosocial well-being in adolescents (strong negative association between MSs and PSWB, c = −0.66), with school-related self-concept acting as a partial mediator (variance of MSs and the three school-related SCs explained 44% of the variance in PSWB).
Wagner et al. (63)	Test of ESH & Expand	Longitudinal	940	Assessed twice at three different time points: starting at 6 years, between 6 and 10 years and from 12 to 16 years	462 M; 478 F	Germany	DCD; ADHD; Language Problems; No DCD	The study supported the ESH by showing that gross motor coordination problems in elementary school were associated with various negative outcomes in adolescence, including persistent coordination problems (OR = 7.99, *p* < 0.001), avoidance of physical activities (OR = 1.53, *p* < 0.05), elevated body mass (OR = 1.78, *p* < 0.05), bonding with sedentary peers (OR = 1.84, *p* < 0.01), emotional problems (OR = 1.73, *p* < 0.05), and conduct problems (OR = 1.79, *p* < 0.05).
Wagner et al. (33)	Provide evidence for ESH	Cross-sectional (Correlational)	70	7.69 years	27 M; 8 F	Germany	DCD; No DCD	The study underscored the role of peer problems in mediating the relationship between motor skills and internalizing/externalizing problems in school-aged children (greater peer problems associated with greater internalizing [*β* = 0.197, *p* < 0.05] and externalizing problems [*β* = 0.405, *p* < 0.01), indicating that interventions targeting peer relationships may help mitigate behavioral problems associated with motor impairments.
Waszczuk et al. (64)	Provide evidence for ESH	Cross-sectional (Correlational)	2,518	25.30 years	856 M; 1,662 F	UK	DCD; Anxiety; Depression	Coordination difficulties, anxiety, and depression had significant familial (50% for coordination difficulty) and non-shared environmental influences, with a substantial overlap of familial liability for these problems (familial influences accounted for over half of the phenotypic correlations between coordination difficulty and internalizing symptoms).
Wilson et al. (34)	Provide evidence for ESH	Cross-sectional (Correlational)	532	5.42 years	291 M; 241 F	Australia	Normative sample	Social skills fully mediated the relationship between motor ability and internalizing symptoms (indirect effect = .06, *p* = .003) in typically developing children. The direct pathway from motor ability to internalizing symptoms was non-significant [*β* = −0.10, 95% CI (−0.22, 0.02), *p* = 0.097] when social skills were considered as a mediator.
Zwicker et al. (11)	Provide evidence for ESH	Inductive Realistic Approach	13	9.92 years	10 M; 3 F	Canada	DCD	Children with DCD faced significant challenges in their daily lives, affecting their physical, psychological, and social quality of life domains, as revealed through thematic analysis of semi-structured interviews.

^a^ Environmental Stress Hypothesis (i.e., Test, review, commentary, referenced, extension). “Provide evidence for ESH” pertains to studies that were not explicitly designed to test the ESH. In contrast, “Test of ESH” refers to studies that explicitly aimed to test specific pathways delineated by the ESH framework.

We first categorised findings, mechanisms, and ideas into two groups: (1) those that relate to and/or support the original ESH and (2) those that consider new dynamics not originally seen in the ESH. For the studies that fit within the original model, we allocated them to their respective pathways. For the studies that did not fit, we developed general themes and sub-themes for grouping, which we later used to inform the conceptualisation of potential additions to the ESH (i.e., new pathways). This analysis helped us identify similarities and differences in findings and to explore interactions of different variables that did not conventionally fit in the original ESH. Due to the complexity of the phenomena of interest and the interdependence of these pathways, some studies were grouped into multiple sub-themes, reflecting the intricate nature of the relationships contributing to increased internalising problems. Data from both qualitative and quantitative studies were integrated to provide a comprehensive understanding of the ESH framework's contributions, with detailed analysis and categorization discussed in the Results section.

## 3 Results

### 3.1 Characteristics of selected articles

**Figure 2:**
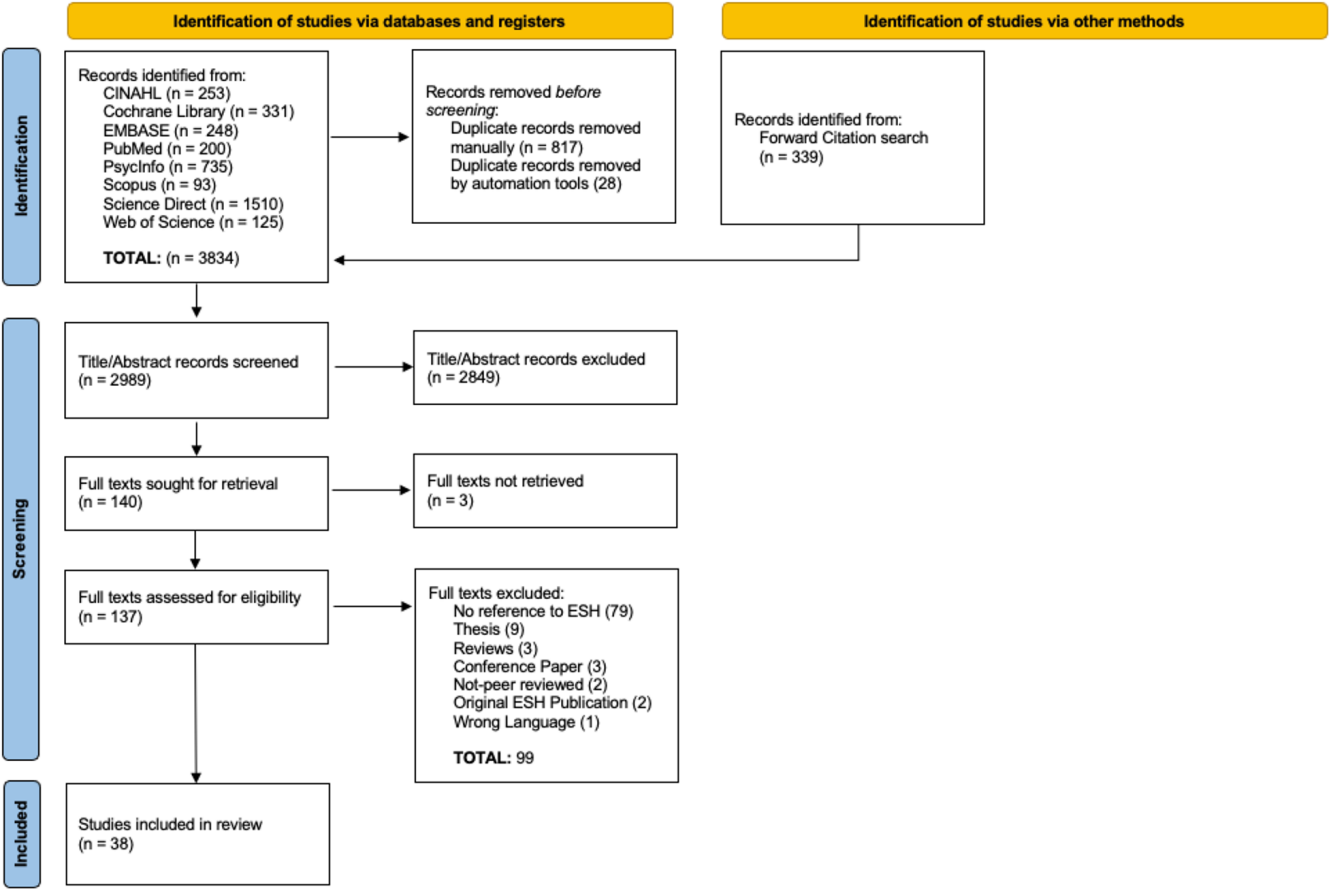
Study identification and selection process.

A total of 38 articles were selected for inclusion in this review. Two reviewers (NE, JB) were able to achieve an inter-examiner agreement rate of over 85%, disagreeing on the inclusion of 21 articles out of 140 articles that were fully screened, which were later resolved following a discussion between reviewers. Figure 2 outlines the process. Thirty-two of the 38 selected articles were studies that directly assessed one or more pathways of the ESH, or were closely related to the ESH (e.g., the ESH was cited as an explanation for findings). The other 6 articles were not empirical but provided pertinent theoretical and analytical insights that helped expand the model including: 1 international clinical practice recommendations guideline (4), and 5 practical and/or theoretical reflection pieces (3, 56, 38, 39, 42). Twenty-five of the 32 empirical studies used cross-sectional analyses to evaluate the associations of the proposed pathways in the ESH, whereas 3 had a longitudinal design (27, 43, 63), 3 had an experimental design (52, 53, 55) and 1 used a qualitative interview design (11). Concerning sampling, 16 studies had a unique sample, while the other 16 studies used samples derived from shared data sources. Of the studies that used a shared sample, 14 looked at cross-sections pulled from larger longitudinal studies, with data from the CATCH (65) the most commonly used data source of these studies (*n* = 4). Notably, each study that shared a data set assessed unique variables and pathways of the ESH mitigating any redundancies. Of the 32 included titles, 27 employed samples of children and or adolescents, whereas 5 used adult samples (20, 44, 47, 59, 64). Only 17 studies examined sex differences or included it as covariate related to the relationship between poor motor skills and internalising problems. Further, community samples were the overwhelming sample type, used in 26 of the studies. This included 13 studies that only evaluated a normative sample (i.e., typically developing), 6 studies sampled individuals considered to be at risk for DCD (probable DCD—pDCD (26, 37, 45, 46, 48), 6 samples of individuals formally diagnosed with DCD (25, 43, 44, 54, 59, 60), 3 samples of individuals with ADHD (25, 41, 59), 1 sample of individuals with ASD (59), 1 sample of individuals with global developmental delay [GDD; (60)] and 3 studies that had a sample of individuals with multiple comorbidities (e.g., individuals with ASD, DCD and ADHD (25, 59, 60). In contrast to the 26 studies that used community samples, 6 studies explicitly used a clinical sample, where participants were recruited via paediatric therapists, clinicians, or occupational therapists. These populations included children with ADHD (50) and children with DCD (11, 33, 52, 53). One study (61) recruited children from a school for children with extreme emotional and behavioural difficulties, where these children were excluded from the mainstream schooling system. Regardless of sampling procedures, all the included studies help to inform the complex relationships between poor motor skills, internalising problems, and other relevant variables across the spectrum of motor competency as the reviewers originally intended for.

### 3.2 Quality of studies

Quality appraisal of the identified studies were performed on 31 studies, excluding the non-empirical studies as previously mentioned [e.g. (4)], and Zwicker et al. (11) due to the subjectivity of the studies qualitative design. The two individual appraisers (JB & NE) were able to achieve an excellent inter-rater variability agreement (*κ *= 0.98), where both appraisers were able to reach a unanimous agreement for each discrepancy following a discussion. Ninety per cent of the studies were rated as high quality (scores 8 and above), 10% were rated as moderate quality (scores 5–7) and none of the included papers were rated as low quality (scores 4 and below). Where the included studies struggled the most (*n* = 26) was with providing justification for their respective sample sizes. Other issues, although not as prominent, were studies’ (*n* = 11) samples not being sufficiently representative of their targeted population, and studies (*n* = 8) not adequately controlling for potential confounds.

### 3.3 Evidence of a direct relationship between poor motor skills and internalising problems

The ESH posits that individuals with poor or impaired motor skills, such as DCD, have a greater propensity for experiencing internalising problems [e.g., anxiety disorders (22)]. Fourteen studies show support for this direct relationship, 8 (26, 32, 33, 46, 48–50, 54) of which show a direct effect for the relationship—with the average effect size, calculated as the average of standardized regression coefficients (β) or path coefficients (c’) from these studies, being approximately −0.90, suggesting a large negative relationship between poor motor skills and internalizing problems. Corroborated, by example, Mancini, Rigoli, Heritage, et al. (32) who found moderate negative correlations between motor skills and anxiety (*r* = −0.32) and depression (*r* = −0.33).

The other 6 studies (25, 43, 44, 47, 58, 63) provide support for this relationship through significant group differences in internalising symptoms between those with DCD/motor deficits and typically developing controls. For example, Hill & Brown (44) found large effects for state anxiety (partial *η*^2^ = 0.228), trait anxiety (partial *η*^2^ = 0.355), and depression (partial *η*^2^ = 0.244), with the DCD group showing higher symptom levels compared to typically developing adults.

The overwhelming evidence of the relationship between poor motor skills and internalising problems was seen through indirect/complex pathways, explored in the subsequent section.

### 3.4 Evidence of an indirect pathway: multiple indirect pathways for poor motor skills and internalising problems

The ESH emphasises that DCD can trigger a cascade of secondary stressors that can increase the risk of internalising problems (5, 22). All 32 of the empirical studies yielded insights surrounding multiple indirect pathways of this model. Thirteen of the studies explicitly tested multiple pathways of the ESH, citing the model directly as inspiration for their respective studies. Whereas the other 19 of the included studies provided pertinent insights for multiple pathways, that when extrapolated, further elaborated dynamics of the ESH. The following sections (3.5 and 3.6) will expand upon these findings, specifying the role in which they helped us better understand the framework of interest.

### 3.5 Findings based on the original model

#### 3.5.1 DCD and secondary stressors

Cairney et al. (22) highlight interpersonal conflicts (e.g., with peers, teachers and parents) as secondary stressors that can impact the relationship between poor motor skills and internalising problems. The current review identified 12 studies that provide evidence for a pathway of poor motor skills, interpersonal conflicts, and internalising problems. For these studies interpersonal conflicts were primarily understood through *peer relationships*, which broadly encompassed factors of peer problems, popularity, best friendships and bullying. Four key studies (27, 33, 40, 49) supported a mediation effect of peer problems on the relationship between motor deficits and increased internalizing symptoms in children and adolescents. Gasser-Haas et al. (40) show the effect between poor motor skills and internalising problems became non-significant (*ß* = −0.26) when peer problems was included as a mediator (i.e., peer problems mediated the relationship between poor motor skills and internalising problems).

Other studies found a moderation effect of best friendships (59), qualitative evidence supporting the role of bullying and victimisation (11), complex relationships between poor motor skills and various peer relationship contexts (41, 50, 55, 62) and sex specifics surrounding the relationship between poor motor skills, peer problems and internalising problems—where for example, Harrowell et al. (43) show that males with DCD experienced greater peer problems, contrasting evidence from Wagner et al. (63) who found that the mediating role of peer problems on the relationship between poor motor skills and internalising problems was more prominent for females.

#### 3.5.2 DCD, personal resources, and social resources

*Personal resources* and *social resources* were proposed as potential buffers against stressors within the ESH (22). The model highlights personal resources through: (1) self-concept (e.g., self-esteem, self-competence, mastery and self-efficacy) and (2) social competence (i.e., social skills), and social resources through social support (i.e., either from parents or peers).

Pertaining to *personal resources*, we identified 13 studies that support the influence of these types of resources. Firstly, we found 9 studies that yield insights surrounding the effects of self-concept domains on the relationship between poor motor skills and internalising problems. Specifically, Li, Kwan, et al. (26) and Mancini et al. (27, 49) demonstrated mediation pathways involving various self-concept domains. Mancini et al. (27) report that perceived physical competence partially mediated the relationship between poor motor skills and internalizing problems at an 18-month follow-up [b = 0.01, 95% CI = (0.040–0.002)], where Li, Kwan, et al. (26), found a similar result, but with a more pronounced mediation effect of global self-worth for females. Additionally, Viholainen et al. (62) found that self-worth accounted for significant variance in internalising symptoms for those with poor motor skills. Two studies identified more complex pathways. Rigoli et al. (57) found a mediation pathway where motor coordination influenced self-perceptions (*r* = 0.55), which in turn affected emotional functioning (*r* = 0.55), ultimately driving depression (*r* = 0.07) and anxiety (*r* = 0.39). Similarly, Li et al. (47) demonstrated that poor motor coordination increased secondary stressors, negatively impacting self-concept (*β* = −1.950, *p* < 0.001), leading to higher internalising problems (*β* = −0.741, *p* < 0.05). Other evidence of a relationship between motor coordination, self-concept, and internalising problems was seen in Harrowell et al. (43) who found a significant correlation between DCD and self-esteem, Zwicker et al. (11), who offered qualitative insights on the link between self-esteem, negative emotions, and motor problems, and Noordstar et al. (53) who showed that interventions targeting poor motor skills could improve self-competence domains.

Secondly, we found 6 studies who support a pathway via *social skills*. Two studies (34, 43) provide direct evidence that social skills mediated this association. Wilson et al. (66) showed social skills fully mediated the relationship in pre-primary children (*p* = 0.003), where those with greater social skills exhibited lower internalising symptom levels, which was similarly shown in Harrowell et al. (43) who show mediation effects of social communication and social skills between DCD and internalising symptoms. Other studies provide further insights where one study found that teachers reported that poor motor skills significantly predicted higher levels of social problems for their students (41), another study found evidence in their interviews directly from children with motor impairments how their unique social skills struggles contributed to negative emotions and internalising symptoms (11), and 2 other studies (59, 60) identified differences in social skills and social competencies across DCD diagnostic groups. Tal-Saban et al. (60) showed more pronounced social skills deficits in children with DCD compared to those with just GDD, while Tal Saban & Kirby (59) found that adults with DCD had similar levels of empathy compared to typically developing adults, but their past social difficulties may have been due to external factors.

Regarding social resources, we identified 6 studies that show the influence of one's *social support network* on the primary relationship of interest. Three of these studies (20, 32, 47) provide direct evidence of social support as a mediator, where (20) for example, show a significant mediating effect of perceived social support on the relationship between motor proficiency and internalising symptoms (*p* = 0.039). While Harrowell et al. (43) and Zwicker et al. (11) provide support by showing those with poor motor skills reported worse social support. Wagner et al. (63) added further insight surrounding behavioural patterns in those with poor motor skills, showing that these individuals had a greater tendency to seek social support from sedentary peers.

#### 3.5.3 DCD, physical inactivity, obesity, and mental health

Cairney et al.'s (22) ESH model includes *physical inactivity* and *obesity* as various pathways affecting the relationship between DCD and internalising problems.

Concerning physical inactivity, the evidence was mixed. Li, Graham, et al. (46) show a significant moderated moderation effect through physical activity and self-worth. Specifically, physical activity moderated the relationship between poor motor skills and self-worth (coefficient = 0.052, *p* < 0.05), and self-worth then moderated the relationship between poor motor skills and internalizing problems. This suggests that higher levels of physical activity can enhance self-worth, which then reduces internalising problems in children with DCD. Bulten et al. (37) reported a moderating effect, with motor coordination problems predicting higher anxiety/depression at higher levels of sedentary behaviour (significance at 0.27 h/day above the mean). Li, Kwan, et al. (26) found that physical activity mediated the relationship between motor skills and internalizing problems. In contrast, Li et al. (47, 48), Hill & Brown (44), and Medeiros et al. (51) did not find significant mediation effects through physical activity levels.

Similarly, for obesity, the findings were mixed. Li, Graham, et al. (46) found that children with probable DCD had significantly higher BMI scores compared to TD children (*p* < 0.05, medium to large effect size), along with more internalising problems, worse motor coordination, lower levels of physical activity, and lower global self-worth. Another study by Li, Kwan, et al. (26) showed BMI acted as a mediator in sequential pathways involving physical activity and self-worth (indirect effect = −0.012, *p* < 0.01). James et al. (67) demonstrated indirect effects for BMI on physical fitness outcomes in those with poor motor skills, suggesting a new potential pathway. Rigoli et al. (20) found that BMI influenced internalising symptoms through the mediator of physical self-worth (effect size = *β* = −0.108, *p* = 0.039). Conversely, Li et al. (47, 48) and Medeiros et al. (51) did not find a significant mediating or moderating effect for BMI on the relationship between poor motor skills and internalising problems.

### 3.6 Findings based on new proposed variables

As previously mentioned, this review identified novel factors from the literature that extend the original ESH model. In order to clearly organise and report these findings, this section groups them based on their overarching theme and derivative subthemes. Inferences on how these factors relate to and/or further develop pathways of the ESH are explored more comprehensively in the Discussion.

#### 3.6.1 Poor motor skills, interpersonal variables and prosociality

Originally the ESH proposed interpersonal conflicts and social resources as influencing factors affecting the relationship between DCD and internalising problems. This review, however, identified additional variables with respect to interpersonal phenomena, prosociality and the ESH. *Interpersonal phenomena* in this context broadly relate to one's interactions with others, including their behaviours and perceptions towards others, which also align with and extend previously reported interpersonal stressors, and personal and social resource buffers. We identified 14 studies that add to this more comprehensive theme of interpersonal phenomena across 3 derivative sub themes.

(1) Studies evaluating *externalising problems*: This sub theme pertains to behaviours reflective of antisocial conduct, including rule breaking behaviours, aggressiveness, disruptiveness, and oppositionality. Nine of the included studies provide insights surrounding an influence of externalising problems in relation to poor motor skills and internalising problems. Medeiros et al. (51) and Wagner et al. (63) provide direct evidence that externalising behaviours mediate this relationship, with Wagner et al. (63) suggesting a cascading pathway in elementary school children from motor impairments to conduct problems to emotional distress over time. They found that their participant's with gross motor coordination problems had a significantly higher risk of developing conduct problems in adolescence [OR = 1.79, 95% CI (1.03–3.11), *p* < 0.05], which were then predictive of a greater likelihood of experiencing emotional problems and internalising difficulties during adolescence [OR = 1.73, 95% CI (1.02–2.93), *p* < 0.05]. Other findings included complex interactions between externalising problems, peer problems, and internalising problems in those with poor motor skills (33, 50), evidence that poor motor skills correlated with both internalising and externalising problems (45), and various insights surrounding behaviours like aggression and disruption contributing to negative social interactions—critical risk factors for internalising symptoms (41, 55, 58, 62).
(2) Studies evaluating *prosocial behaviours*: This relates mainly to empathetic behaviours, selfless acts, and a person's willingness to help others, which were examined in 5 of the studies. While the original model focuses on developing social skills, which prosocial behaviours are naturally connected to, these prosocial behaviours are more intrinsically linked to the individual, requiring special considerations surrounding the motivations and psychological profile of the individual (68–70), pp. 93–94). In other words, social skills represent the proficiency of the behaviour which leads to either positive or negative social outcomes, where prosocial behaviours reflect more of the internal motivations behind the behaviours. Two studies (59, 60) found significant differences in empathy between groups. Tal Saban & Kirby (59), for example show that participants with DCD or DCD combined with one or more comorbidities, such as ASD and ADHD, scored significantly lower in empathy [F(8,257) = 9.98, *p* < 0.001, *η*^2^ = 0.162]. Thus, the authors’ suggested that children with DCD, and/or with additional comorbidities have greater difficulties in understanding and responding to others’ emotions, subsequently leading to other adverse psychosocial consequences. Viholainen et al. (62) found that better motor skills associated with more positive peer interactions and higher levels of prosocial conduct. With further elaboration seen in Wilson et al. (34) who report that poorer motor skills were additionally linked to lower perceived social competence and fewer prosocial behaviours in children. On the other hand, while Piek et al. (55) were to able show that their play-based motor skill intervention program targeting motor skills and social skills in younger children improved prosocial behaviours, the program did not explicitly reduce internalising problems for these children.
(3) Studies that *evaluate family environments*: This sub-theme focuses on variables relating to a person's family dynamic and upbringing. Specifically, home life situations that can affect one's relationship and attitudes towards their own motor problems, along with opportunities for treatment and other remedial resources. Taylor et al. (61) found that family disruptions and upsets significantly exacerbated the negative effects of motor skill deficits on internalising symptoms. Children from less stable home environments exhibited higher levels of internalising symptoms when struggling with motor impairments. Similarly, the longitudinal study by Harrowell et al. (43) reported that adolescents with DCD who had more supportive family environments were less likely to develop severe mental health complications compared to those from less supportive backgrounds, as they specifically found parental mental health and higher socioeconomic status to be significant protective factors in their analyses.

#### 3.6.2 Poor motor skills, intrapersonal characteristics, and executive functions

A prominent theme was identified in the literature: studies evaluating one's *intrapersonal characteristics* and executive functions as facilitators for increased internalising troubles in individuals with poor motor skills. This theme highlights the influence of predominantly inward, or internal, stressors that one may experience. It encompasses aspects related to one's self-regulatory, and cognitive abilities. Specifically, their capacity to manage emotions, control impulses and perform goal-directed behaviours. We identified 16 studies that report on this overarching construct which we then subdivided into 5 subthemes.

(1) Studies that investigate *affective executive control*: This entails cognitive functions associated with inhibitory control of hyperactivity and impulsiveness, cognitive flexibility, ability to effectively switch between tasks and emotional coping strategies. Thirteen of the included studies examined factors relating to this. Omer & Leonard (54) reported that executive function (EF) difficulties, particularly in behavioural regulation aspects (e.g., inhibition and emotional control), mediated the relationship between DCD and internalising symptoms [indirect effect = 14.20, 95% CI (7.12–21.02)], with the direct effect becoming non-significant when accounting for this mediation (path c’ = 5.08, *p* = 0.201). Several studies (41, 50, 57, 62) provided information surrounding various complex pathways through affective executive control not originally indicated in the ESH. Rigoli et al.'s (2012) model, for example, show self-regulation abilities impacting emotional functioning which later manifested in internalising problems. Other findings included significant group differences pertaining to emotional reactivity and executive control challenges for those with poor motor skills compared to controls (25, 48, 58–60), improvements in hyperactivity following motor skills training (55), qualitative data linking motor deficits to compromised emotional coping (11), and neurological markers for motor and emotional dysfunction (61).
(2) Studies that examine *metacognitive executive functions*: This refers to evaluations of working memory, verbal comprehension, perceptual reasoning, and processing speeds (i.e., functions that could be indicative of underlying learning troubles). We identified 5 studies that align with this sub theme. Omer & Leonard (54) and Rigoli et al. (57) demonstrated that metacognitive deficits, manifesting as challenges in areas like reading comprehension and learning, further contributed to higher anxiety and depression among those with compromised motor skills. Omer & Leonard (54) show a mediation effect of metacognitive EF on the relationship between DCD and internalising symptoms [indirect effect = 14.20, 95% CI (7.12–21.02)]. Taylor et al. (61), Wilson et al. (66) and Zwicker et al. (11) further explain a pathway where deficits in metacognitive functions for those with poor motor skills can negatively affect academic achievement, lowering self-esteem, ultimately leading to negative internalising outcomes (i.e., poor motor skills → deficits in metacognitive EF → academic difficulties → lower self-esteem → more psychological distress).
(3) Studies that investigate *sensory processing*: Sensory processing in this case refers to studies that review an individual's sensory profile, as well as their sense of agency (i.e., subjective experience of initiating and controlling their own actions). Nobusako et al. (52) found that children with DCD exhibited an altered time window for sense of agency, which in turn, positively correlated with depressive symptoms (*r* = 0.475, *p* < 0.05). While Tal-Saban et al. (60) found that children with combined DCD and GDD were prone to sensory-processing deficits compared to GDD, along with significant group differences in internalising problems.
(4) Studies that consider *neurological and somatic dysfunction*: This sub theme refers to studies that examine constructs relating to an underlying cortical and/or nervous system dysfunction. Four studies elucidated insights surrounding these difficulties and their relationship to poor motor skills and internalising problems. Two studies considered the impact of chronic stress exposure (i.e., subjective experiences of constantly feeling stressed from daily activities and life changes). Li et al. (47) found secondary stressors, such as chronic stress, significantly mediated the relationship between poor motor skills and internalising problems (*β* = 0.460, *p* < 0.001). Findings that further related to the evidence seen in Zwicker et al. (11) who's participants highlight the significant effects of daily chronic stress resulting from their poor motor skills, and how it detrimentally impacts their mental health (e.g., increased depression/anxiety). Along with this, Li et al. (48) provide insights surrounding somatic complaints (i.e., consistent feelings of physical symptoms like aches, pains, fatigue, or general malaise). The authors found significant group differences in somatic complaints (*p* < 0.05) between children with DCD and typically developing peers, potentially highlighting another influence towards increased internalising problems. Lastly, Taylor et al. (61) showed substantial evidence concerning primary reflex persistence, characterised as neonatal autonomic reflexes (e.g., Moro reflex) that persist into later stages of development (i.e., late-childhood and adolescence). The authors found this form of reflex motor dysfunction, which is often indicative of cortical and/or nervous system dysfunction, was more present in those with severe emotional and behavioural problems, linking neurological markers to motor impairments.
(5) Studies considering *global relationships* (i.e., attachment styles): This sub theme encompasses intrapersonal biases relating to attachment patterns in relationships (i.e., anxious and avoidant tendencies). These tendencies were considered by Li et al. (47) who found poor motor coordination significantly related to both higher psychological distress (*β* = 0.460, *p* < 0.001) and negative attachment styles. Anxious attachment mediated the relationship between motor coordination problems and internalizing difficulties (indirect effect = 0.097, *p* < 0.05), as well as mediation from avoidant attachment (indirect effect = 0.101, *p* < 0.01). Moreover, the authors show that these children were more likely to experience anxious attachment (*β* = −0.193, *p* < 0.01), increasing sensitivity to social rejection and the risk of internalising problems. They also found that perceived social support mitigated the impact of insecure attachment on internalising symptoms (*β* = −0.960, *p* < 0.001).

#### 3.6.3 Poor motor skills, health behaviours and other factors

While the original ESH model considers the influence of physical inactivity and obesity, the current review identified several additional variables which highlight health and lifestyle factors. This includes the following 3 sub themes.

(1) Studies that measure *fitness levels*: Fitness level measurements were of interest for (45). In their study, they show a unique mediation of internalising problems on the relationship between children at risk for DCD and their physical fitness levels; measured both through the *Bruce Protocol* (71) total test time [95% CI = (−7.54 to −0.92), psie = –0.04], and through the *Wingate* test (72); peak power [95% CI = (−1.39 to −0.12), psie = –0.02] and mean power [95% CI = (−1.46 to −0.19), psie = –0.03]. These findings provide nuance to the ESH, highlighting how internalising problems connect to fitness levels, which can, plausibly, further decrease physical activity levels for a population that already is disproportionately averse to exercise (73, 74).
(2) Studies investigating *sleep problems*: While, Rodriguez et al. (58) did not find significant group differences for sleep problems within the CBCL, Mancini et al. (50) found that poor motor coordination and sleep difficulties predicted increased peer problems, even after accounting for ADHD symptoms and executive dysfunction—highlighting a new potential health metric that could be further examined for the ESH, as sleep difficulties have been previously shown to be linked to internalising problems [e.g., (75)] and decreased executive functions [e.g., (76)].
(3) Studies that evaluate *health related quality of life*: Health related quality of life refers to studies that consider one's overall rating of their physical and psychosocial well-being. Medeiros et al. (51) did not find significant mediation of overall physical health on the relationship between poor motor skills and internalising problems in children. However, compelling evidence from the interviews shown in Zwicker et al. (11) highlight jarring perspectives surrounding the presence of motor difficulties negatively affecting both daily tasks and participation in desired activities, which the participants explained to impact their overall quality of life, physical and emotional states.

### 3.7 Causality

The Environmental Stress Hypothesis suggests a relationship between motor skills and internalising problems, but the evidence supporting this theory is largely based on cross-sectional studies that do not provide causal evidence. While previous experimental studies have supported the hypothesis, directionality and causal underpinnings remain unclear. We identified only 3 longitudinal studies (27, 43, 63), which help to show evidence of temporal precedence, but these studies did not cover all the pathways proposed by the hypothesis. Further experimental and longitudinal research is necessary to establish causality more definitively and to methodically examine the complex pathways in the framework (13, 55, 63, 64).

## 4 Discussion

To better understand the current uses of the ESH, we analysed insights from a total of 38 qualitative and quantitative sources on the topic. Building from Mancini et al. (9), we found 19 additional studies since their review, as well as 7 qualitative works relevant to the phenomena of interest.

The data from this review supports the hypothesis that poor motor skills are associated with increased internalising problems, consistent with conclusions drawn by Mancini et al. (9). This relationship was thoroughly understood as being driven through various complex pathways. Despite variations in the extent to which studies explicitly tested or inadvertently explored multiple ESH pathways, no one study has yet to comprehensively evaluate all pathways, emphasising the necessity for further in-depth investigation.

While studies of DCD primarily focus on children, interestingly, 5 studies considered the relationship of interest in adults. Issues with poor motor function, as well as internalising problems, are believed to persist into adulthood (23, 43, 77). Although understanding the development of the ESH in younger populations is important, older cohorts possess a greater capacity to articulate their emotions and symptoms with precision. These nuanced insights, shaped by lived experiences, can provide valuable perspectives on the phenomena at hand; particularly important as the implications of poor motor competence and stressors (e.g., peer problems) evolves with and dynamically shifts with environmental demands (41). Therefore, the intricacies of how the interplay between poor motor skills and internalising problems matures throughout adulthood remain poorly understood and need further investigation.

Similarly, some researchers [e.g., (78)] argue sex to be an individual-level variable that can significantly influence the relationship between poor motor skills and internalising problems. The findings were mixed, where studies such as Mancini, Rigoli, Heritage, et al. (32) did not find a significant moderation effect of sex, while studies such as Bulten et al. (37) found significant sex-differences in their sample, with girls at risk for DCD reporting significantly higher levels of internalising problems compared to the TD groups and rDCD boy group. A possibility for these mixed findings can be that of the studies that examined sex, 4 studies (20, 44, 59, 64) used adult samples. Research suggests that sex differences in internalising problems do not typically begin until puberty, becoming more pronounced over time (79, 80). For instance, Harrowell et al. (43) found older adolescent females with DCD experienced greater emotional distress when compared to age-matched boys with the condition. Green & Payne (42) suggest that this trend aligns with The Dyspraxia Foundation's survey (81), further explaining that females tend to receive diagnoses at later ages, often prompted by a breaking point when existing coping mechanisms no longer suffice. Thus, it is plausible that the mixed findings were predominantly driven by the overwhelmingly young samples, where such interactions are not yet present or discernible. This further reinforces the need for longitudinal investigations to pinpoint crucial developmental periods where divergences in sex emerge.

### 4.1 The original ESH model

As previously mentioned, the findings from this review are largely consistent with the ESH framework. The studies from this review emphasise significant evidence for both a relationship between poor motor skills and internalising problems [e.g., (54)], and for various intermediary pathways influencing this relationship [e.g., (47)].

We identified overwhelming support for secondary stressors (i.e., interpersonal conflicts) within the ESH model. Studies such as Gasser-Haas et al. (40) supported a mediation effect of peer problems exacerbating internalising problems in those with poor motor skills. Researchers like Pfeifer & Allen (82) emphasise the intense visceral emotional responses that peer conflicts alone can cause. This is further supported by the psychiatric literature, which is replete with examples of acute peer rejection paradigms such as Cyberball [e.g., (83–86)] demonstrating stress-induced neural activation in various brain regions linked to predicting future internalising problems [e.g., (87)], including the subgenual anterior cingulate cortex, ventrolateral prefrontal cortex, posterior midline regions, ventral striatum, amygdala and medial orbitofrontal cortex (88–92). Due to this existing precedent, it is unsurprising that children with poor motor skills, who often face more peer problems, would experience more heightened emotional responses. While there is some evidence supporting a link between emotional dysfunction and motor deficits [e.g., (54, 61)], further research is needed to elucidate if there's a unique mechanistic pathway that leads to a biased and disproportionate stress response to interpersonal conflicts for those with compromised motor abilities.

Cairney et al. (5, 22) cite both personal and social resources as key features within the ESH model. While the exact nature of how these resources influence the relationship between poor motor skills and internalising problems requires further specification (e.g., do they moderate or mediate?), the studies we reviewed provide considerable evidence of their impact. Regarding personal resources, various components of self-concept were shown to mediate the relationship between poor motor skills and internalising problems [e.g., (49)]. This concurs with research [e.g., (93)] showing an inverse correlation between self-concept and negative mental health outcomes such as suicidality, feelings of loneliness and ultimately depression. Adverse psychosocial outcomes that individuals with motor deficits may be more prone to (4, 94). Additionally, the personal resource of social skills was shown to have a significant impact on the relationship between poor motor skills and internalising problems. Where various studies (e.g., Wilson 2013 and Harrowell), show a mediation effect of social skills and social communication on the relationship between poor motor skills and internalising problems. Tal-Saban et al. (60), further explain that motor problems detrimentally affect social engagement from an early age. Children with DCD demonstrated reduced participation in diverse motor skill-requiring social scenarios, including both organised and unstructured peer play and group physical activities. This restricted involvement limits their opportunities to develop and hone their social skills, leading to potential limitations in maintaining their social standing (9, 95).

The resource of social support was additionally reinforced by our findings. Several studies [e.g., (20)] found that perceived social support mediated the relationship between poor motor skills and internalising problems. Research has shown that healthy connections and relationships help mitigate harmful responses to stressors by reinforcing a more benign perception of the circumstance (96, 97). While more research is needed to clearly define the role of social support within the ESH model, the importance of a supportive social network in ameliorating the psychological impact of DCD is evident. Therefore, continued efforts should be directed to improve social competencies for this population, building personal resources and enabling the development of improved social resources.

Physical inactivity and obesity were important factors added to the ESH. The support surrounding their influence on the relationship between poor motor skills and internalising problems was mixed. Li, Graham, et al. (46) show evidence of a pathway between physical inactivity and the relationship between poor motor skills and internalising problems, as well as (20) showing evidence of a pathway of BMI and physical worth. Yet, Li et al. (48) were unable to show a significant effect or pathway for both physical inactivity and obesity. Possible rationale for the mixed findings can include the limited number of studies that tested these pathways, given the relative novelty of the model, and differences in defining motor deficits as noted by Li et al. (48). Moreover, Li et al. (47) suggested that low levels of physical activity for both their motor deficit and TD group could be symptomatic of a larger floor effect at the participants’ age, making it difficult to detect a mediational effect. The authors also rationalised their lack of findings surrounding BMI to their small sample size. Despite the present mixed findings, the general evidence of increased physical activity and decreased obesity levels being a net positive for mental health is overwhelming [e.g., (98, 99)]. Researchers should continue to flesh out these pathways and develop a better understanding surrounding the relationship between obesity and physical inactivity to clarify their role within the ESH.

While the studies in this review show overall support for the ESH, there are several considerations within the literature that warrant an expansion of the model. First, the applicability and viability of the ESH to contexts of impaired motor function beyond DCD. Second, the acknowledgment of the stress implications of novel secondary interpersonal stressors (i.e., externalising problems) within the framework. Third, the consideration of the potential influence of intrapersonal struggles as secondary stressors (e.g., sensory processing and attachment styles). Fourth, the addition of novel stress-buffering effects of personal resources (e.g., prosociality). Fifth, the integration of environmental contexts into the social resource pathway. Lastly, the broadening of physical activity to poor health practices and negative health outcomes beyond obesity.

**Figure 3:**
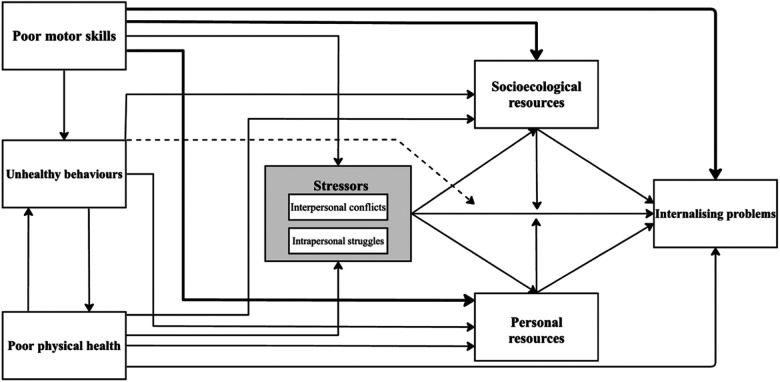
Updated ESH.

In response to these insights, we updated various components of the ESH (see Figure 3). In our updated model, the arrows depict different pathways leading to internalising problems. Poor motor skills are identified as a primary stressor that can either directly cause internalising problems or trigger secondary stressors that intensify these problems. Secondary stressors are depicted in the dark grey box, which now includes both interpersonal conflicts and intrapersonal struggles. The updated ESH incorporates stress buffers in the form of socioecological and personal resources that can mitigate the effects of stressors. Our model also updates the pathway of physical inactivity to encompass unhealthy behaviours and broadens the pathway of obesity to poor physical health. Both new pathways can impact intermediary pathways and includes bi-directional arrows representing the feedback loop of their reciprocal relationship. Additionally, unhealthy behaviours are shown with a broken arrow to indicate their potential influence on the relationship between secondary stressors and internalising problems.

Further details of these modifications are provided in the section below. It is important for us to emphasise that, like its predecessor, our updated model serves as a heuristic framework for better understanding the relationship between poor motor skills and subsequent internalising problems and should be treated accordingly.

## 5 Modifications to the ESH

### 5.1 Modifications to the ESH I: framing poor motor skills as a primary stressor

The original ESH model was framed around DCD; motivated by the evidence showing the stress implications of DCD on daily self-care activities, schooling experience and performance, and peer relations (2, 4, 100, 101). The literature, however, has expanded to evaluate poor motor skills more broadly, encompassing subjects who experience significant motor deficits without formal diagnoses and other neurodevelopmental disorders affecting motor skills. It seems reasonable therefore to conceptualize poor motor skills as a broader, more inclusive category than DCD *per se*.

### 5.2 Modification to the ESH II: broadening the construct of secondary stressors

Similar to Pearlin's model, Cairney et al. (22) argue that the relationship between the primary stressor (i.e., poor motor skills) and internalising problems can be mediated by various secondary stressors. In the case of the original ESH model, the effect of these secondary stressors were explicitly articulated through interpersonal conflicts: conflicts between the individual, their peers, guardians, and educators as a function of their diagnosis. Compelling findings from the literature prompt for further categorisation of these secondary stressors into interpersonal conflicts, and intrapersonal struggles.

#### 5.2.1 Poor motor skills and secondary interpersonal stressors

The findings in this review substantiate the intricate interpersonal pathway of peer relations outlined in the original ESH [e.g., (27)]. We, however, found several studies introducing novel mediating interpersonal variables which can help to drive the understanding surrounding the correlation between poor motor skills and internalising problems.

A distinct perspective emerges from 9 studies that have included externalising problems, often referred to as antisocial behaviour, within the ESH framework. Externalising problems are defined as conduct-related challenges such as aggressiveness, disruptiveness, and oppositionality. Impulsive, aggressive, and disruptive behaviours can compromise social interactions, consequently driving peers away (102). Those with poor motor skills, such as those with DCD, are at greater risk for exhibiting externalising problems [e.g., (33, 45)]. One of the included studies of this review in particular, Medeiros et al. (51), found that the relationship between poor motor skills and internalising problems was solely mediated via externalising problems. More research is needed to corroborate these findings, and to establish the viability of externalising problems as a pathway and to understand their precise role within the ESH.

#### 5.2.2 Poor motor skills, stress, and intrapersonal struggles

The understanding of mental health outcomes, such as internalising problems, requires one to understand the intersectionality of identities, subjective stressors, and other personal syndemic factors (i.e., the interaction of multiple coexisting conditions) driving lived experiences (103). Insights from the reviewed papers suggest that the ESH framework can be further enriched by considering secondary intrapersonal stressors.

Intrapersonal stress from sensory-processing differences was a unique consideration within the reviewed titles (52, 60). Beyond just immediate motor challenges, individuals with DCD can demonstrate significant sensory differences in visual-spatial processing, proprioception, hearing and vestibular function (4, 104–106), which is consistent with neuroimaging studies that show white matter abnormalities in brain regions such as the corticospinal tract, posterior thalamic radiation, intraparietal sulcus and parietal subregion of the corpus callosum (36, 94, 107, 108). Further, there is evidence of significant sensory processing abnormalities seen in affective disorders [e.g., depression and anxiety; (109–114)]. Remarkably, Nobusako et al. (52) show a bidirectional relationship between movement problems, depressive tendency and a sensory profile differences (i.e., delayed sense of agency). Considering these findings, it is plausible that these disparities, often seen in children with poor motor skills, could lead to heightened intrapersonal stress due to potential misinterpretation or misprocessing of sensory inputs.

The implications of metacognitive executive functions were a novel secondary stressor considered by 5 studies in this review (32, 34, 54, 57, 61). Children with DCD, absent of co-occurring attentional deficits (i.e., ADHD), experience impaired daily planning, academic performance, memory, motor skill automation, and dual-task control (42, 115–119). These findings are consistent with group-level neuroimaging results showing under-activation in brain areas such as cerebellar peduncles, thalamic radiations, corpus callosum, and corticobulbar and corticospinal tracts (66), though these do not translate one-to-one with behavioural outcomes. This is particularly alarming, as not only are these deficits reported to be quite stressful phenomenologically (i.e., subjective experiences (11); such functions have been shown to accurately predict internalising trajectories (120, 121). Therefore, our findings suggesting a potential pathway in which deficits in metacognitive executive functions can induce a sense of internal stress, stemming from perceived inefficiencies in understanding, processing, and planning tasks, which can further instigate the relationship between poor motor skills and internalising problems.

In contrast to metacognitive executive functions, 17 of the included studies examined the intrapersonal stress from affective executive control. These functions are crucial for motor tasks, as they enable adaptive shifts, strategy updates, and goal-directed behaviours in dynamic settings (122). Affective executive control is also associated with predicting future internalising problems (120, 123–127). Omer & Leonard (54), argue that maladaptive affective executive control can be stressful for the individual via symptoms like persistence of negative thoughts [e.g., (128–130)] stress that individuals with DCD may be more prone to as they have been shown to struggle with inhibiting and shifting attention away from negative emotional stimuli [e.g., (128, 130)]. Deficits in this domain can exacerbate intrapersonal stress, compounding the impact on motor performance and contributing to worse mental health outcomes due to difficulties in emotion-driven decision-making or adapting to emotionally charged situations.

Lastly, the ESH framework can be enriched by considering the significant role of neurological and somatic dysfunctions as secondary intrapersonal stressors. Chronic stress exposure, resulting from daily activities and life changes, was seen to significantly mediate the relationship between poor motor skills and internalising problems, as evidenced by Li et al. (47) and Zwicker et al. (11). For many children with significant motor problems, such as DCD, basic daily tasks such as dressing, using utensils, handwriting, and physical play can—be it from their motor skill deficits or the complexities of navigating their unique cognitive profiles—culminate in chronic stress exposure (2, 94). The extent of subjective stress from these difficulties is often hard to gauge, as some problems can be subtle and are often overlooked by overseeing figures and even in diagnosis (4, 131–133). Whether or not individuals with DCD are exposed to more stressors in their environment because of their impairments, or if they mechanistically have a greater sensitivity to stress as a function of their disorder remains unclear. Additionally, somatic complaints such as aches, pains, and fatigue (48) and the persistence of primary reflexes indicative of cortical dysfunction (61) highlight how physical manifestations of stress can drive internalising problems. These findings suggest that underlying neurological issues and chronic somatic complaints can intensify the stress experienced by individuals with poor motor skills, thereby contributing to higher levels of internalising problems. This constant internal tension can potentially magnify the internalising issues associated with poor motor skills.

### 5.3 Modification to ESH III: updating resource buffers

The ESH extends Pearlin's (24) framework by highlighting personal resources through aspects like self-concept and social competence, and social resources through support from parents or peers (22). In the current review, 13 studies (e.g. (60), evaluated the contributions of personal resources, and 6 [e.g., (20)] studies that looked at the effects of social resources. While the support is substantial, recent research suggests a need to address interconnected buffering resources more comprehensively. In our model, personal resources address psychological and emotional stress buffers related to mental well-being. Socioecological resources, on the other hand, focus on external stress buffers, like systemic, socio-cultural, and physical factors.

#### 5.3.1 Poor motor skills and personal resources

Personal resources within the original ESH were largely conceptualised through self-concept and social competence. It is, however, important to recognize the broader scope of skills, beliefs, and individual traits that assist in stress management. Within the positive psychology literature, optimism correlates with enhanced psychological well-being, reduced susceptibility to infections, faster recovery from illness, and more favourable disease trajectories (134–138). Likewise, a sense of control over life circumstances is a strong predictor of superior psychological and physical health, even including lower incidence of coronary heart disease (139–141). Moreover, the concept of resilience, which refers to the ability to recover from or adapt to adverse conditions is also a promising a construct that could be added to personal resources. Resilience is associated with improved emotional regulation and stress buffering, improving psychosocial outcomes such as internalising problems (142, 143), a resource that could be crucial in mitigating internalising problems for those with poor motor skills. Researchers should continue to use the ESH to explore other personal resources to address internalising problems, this in turn will help inform remedial and therapeutic practices.

From our findings, there was support for a pathway of prosocial behaviours, including cooperation, empathy, altruism, and positivity [(144, 145), p. 150]. These types of behaviours were included in 6 studies, where Wilson et al. (34), for example, demonstrate that prosocial behaviours mediate the link between poor motor skills and internalising problems. Gandotra et al. (146) emphasise the importance of prosocial behaviour for healthy social development and adjustment of a child. Prosocial behaviours are quintessential for promoting social group dynamics, facilitating greater cohesion and acceptance; as those who act generously as shown to also influence others to behave more generously (147–150). Those with better motor skills are more likely to engage in active play, which often requires a certain level of cooperation and prosociality to participate. Through these more engaged experiences, the individual creates bonds and develops positive attitudes towards peers, which in turn drive more prosocial behaviours (150–152). As previously mentioned, those with poor motor skills often have fewer opportunities to engage with others, reducing their ability to learn about and practice prosocial behaviours. By teaching and encouraging prosocial behaviours alongside social skills, these behaviours provide individuals with another potential pathway to develop and strengthen friendships, offering more resources to combat internalising problems.

Lastly, in our review we found another pathway of personal resources in Li et al. (47) who show that both avoidant and anxious attachments mediated the relationship between poor motor skills and internalising problems. Global relationships, or attachment styles are believed to develop in infancy and persist throughout life (153, 154). These internal biases influence psychological well-being, with secure attachment predicting better psychosocial outcomes (155, 156). Specifically, anxiously attached individuals display excessive reassurance seeking from others, while avoidantly attached individuals maintain emotional distance in their relationships, both of which can negatively impact the quality and experiences of friendship for the individuals (153, 157, 158). Conversely, secure attachments facilitate the development of coping skills, which are important protective factors (159). Ubha & Cahill (158) found that a 10-week intervention aimed at building secure attachments in primary school children led to positive shifts in their behaviours and experiences, improving their social and emotional behaviours. While it was only one study in our review that found a pathway of global relationships influencing the relationship between poor motor skills and internalising problems, the broader psychological literature shows psychosocial improvements from developing secure attachments (160, 161). Further research is needed to replicate and validate the findings from Li et al. (47). However, fostering global relationships is another potential personal resource that could be cultivated in individuals with poor motor skills to improve subjective experiences and relationship dynamics, thereby buffering stressors that contribute to internalising problems.

#### 5.3.2 Poor motor skills and socioecological resources

Drawing from ecological theories, such as Bronfenbrenner's ecological model (162), we argue for a multilevel understanding of the environmental factors that interact with motor skills and internalising issues. These environmental factors can include not only social elements like family and community support but also features of the physical environment, such as safe play spaces and access to health services. Research has consistently shown that socioeconomic conditions, for instance, have an undeniable influence on child motor development [e.g., (163, 164)]. Lejarraga et al. (165), for example, found that psychomotor performance in children was significantly correlated with familial social status and maternal education. Extending this idea further, children with access to better socioecological resources—such as safer neighbourhoods, quality educational and therapeutic programs—are at an advantage in both motor skill development and stress management. Consequently, understanding motor problems and internalising issues requires a holistic lens that not only considers the psychological aspects but also the diverse and unequal socioecological opportunities available to individuals.

### 5.4 Modification to ESH IV: adding poor motor skills, unhealthy behaviours and poor physical health

A key feature of Cairney et al.'s (22) ESH is the modelling of the adjacent constructs of physical inactivity and obesity. While the findings surrounding both physical inactivity and obesity were mixed, requiring further detangling on their respective pathways, our findings additionally suggested other potential pathways via healthy behaviours and other health outcomes potentially affecting the relationship between poor motor skills and internalising problems. Fitness levels were seen to be a measure of interest for James et al. (45) who found a significant interdependence between poor motor skills, internalising problems and physical fitness levels. Further research should explore this pathway, to see how improved physical fitness levels can affect both physical activity levels, intermediatory pathways and ultimately internalising problems. Other findings prompts explorations of pathways through sleep problems [e.g., (50)] and reported health related quality of life (11). In modifying these distinctions, the model enables better capture of a range of behaviours and outcomes reflective of the heterogeneity of having poor motor skills.

## 6 Conclusion

The ESH serves as a cornerstone in understanding the complex relationship between motor skill deficiencies and internalising problems. Our findings support the hypothesis that poor motor skills precipitate increased internalising challenges, predominantly through intricate indirect pathways. Crucial secondary interpersonal stressors, especially peer relationships, emerge as powerful mediators, reinforcing the profound implications of social dynamics on psychological well-being. Our insights also spotlight the crucial buffers against stress, be it personal facets like self-concept or vital social support structures. A notable gap exists in the consideration of how these relationships may manifest in adulthood, emphasising the importance of focusing on older cohorts who offer invaluable insights through their articulated experiences. Equally compelling is the evidence suggesting that sex may play a pivotal role in the ESH framework, with sex differences emerging in adolescence and becoming more pronounced over time. Our analysis of the ESH also noted possible new areas of expansion and refinement. Future research should focus on not only reinforcing the current findings but also on innovating within the dynamic framework of the ESH, ensuring it remains contemporaneous and responsive to evolving understandings of motor skills and internalising problems.
