# Supplementary material for: Paediatric motor difficulties and internalising problems: an integrative review on the environmental stress hypothesis
Source: Front Pediatr. 2024 Aug 2;12:1320338. doi: 10.3389/fped.2024.1320338 (PMC11327034; doi:10.3389/fped.2024.1320338)
Supplement: Supplementary file 1 [file Datasheet1.pdf]

Supplementary Material

Paediatric motor difficulties and internalising problems: An integrative review on the environmental stress hypothesis

Noah Erskine[1*], Jaime Barratt[1,2], John Cairney[1]

[1] University of Queensland, School of Human Movement and Nutrition Sciences, St Lucia, QLD, Australia

[2] Brock University, Faculty of Education, St. Catharines, ON, Canada

***Correspondence:**
Corresponding Author
n.erskine@uq.edu.au

1 Supplementary Tables

Table 1.1. *Search Terms by Database*

Database	Search Terms
CINAHL	(“DCD” OR “developmental coordination disorder” OR motor impair* AND mental health AND “ESH” OR “environmental stress hypothesis” OR “elaborated environmental stress hypothesis” OR “EESH”)
Cochrane Library	(“DCD” OR “developmental coordination disorder” OR motor impair* AND mental health AND “ESH” OR “environmental stress hypothesis” OR “elaborated environmental stress hypothesis” OR “EESH”)
EMBASE	(motor skills OR motor proficiency OR motor coordination OR motor development OR motor ability OR DCD OR developmental coordination disorder OR coordination OR dysp* OR clums* OR awkward OR motor impair* OR movement disorder OR movement problems OR move* AND well-being OR mental health OR anxi* OR depress* OR "stress" OR self* OR psychosocial OR mental competence OR emot* competence OR mental behaviour OR behaviour AND “ESH” OR “environmental stress hypothesis” OR “elaborated environmental stress hypothesis” OR “EESH”)
PubMed/MEDLINE*	(motor skills OR motor proficiency OR motor coordination OR motor development OR motor ability OR DCD OR developmental coordination disorder OR coordination OR dysp* OR clums* OR awkward OR motor impair* OR movement disorder OR movement problems OR move* AND well-being OR mental health OR anxi* OR depress* OR "stress" OR self* OR psychosocial OR mental competence OR emot* competence OR mental behaviour OR behaviour AND “ESH” OR “environmental stress hypothesis” OR “elaborated environmental stress hypothesis” OR “EESH”)
PsycInfo	("DCD" OR "developmental coordination disorder" AND "ESH" OR "environmental stress hypothesis" OR "EESH" OR "elaborated environmental stress hypothesis")
Scopus*	(motor skills OR motor proficiency OR motor coordination OR motor development OR motor ability OR DCD OR developmental coordination disorder OR coordination OR dysp* OR clums* OR awkward OR motor impair* OR movement disorder OR movement problems OR move* AND

	well-being OR mental health OR anxi* OR depress* OR "stress" OR self* OR psychosocial OR mental competence OR emot* competence OR mental behaviour OR behaviour AND “ESH” OR “environmental stress hypothesis” OR “elaborated environmental stress hypothesis” OR “EESH”)
ScienceDirect	("DCD" OR "developmental coordination disorder" AND "ESH" OR "environmental stress hypothesis")
Web of Science	("developmental coordination disorder" AND "ESH" OR "EESH" OR "environmental stress hypothesis" OR "elaborated environmental stress hypothesis")

To be consistent with previous reviews on the topic, study quality was evaluated via a modified critical appraisal skill programme inspired by Mancini et al. (2019). For this paper, the evaluation of study quality was limited to studies that have either experimental or observational research designs. Ineligible papers (i.e., reviews, critiques, and editorials) were flagged and served as commentary surrounding the conceptualisation and logistics of the model. Eligible studies were scored out of 10 items that and rated as either 1 (confirmed) or 0 (unconfirmed). The rating scale consisted of high quality (scores 8 and above), moderate quality (scores 5-7), and low quality (5 and below; Mancini et al., 2019; Wilson et al., 2017). Study appraisals were conducted separately by two independent researchers. Inter-rater variability no greater than 15% (i.e., 85% agreement) was considered acceptable, and any discrepancies in rating was resolved in a meeting between appraisers.

Modifications to the appraisal. As previously noted, this appraisal was designed to be consistent with the appraisal used in an earlier review of the ESH by Mancini et al. (2019). There are, however, several adjustments to be noted. Due to the potential evolution of the ESH, the researchers felt that it would be appropriate to change the language of the appraisal to consider motor problems at large, instead of singling in on DCD. For similar reasons, the language was changed to be open to any age group, instead of limiting it to children.

Summary:

- 1 = confirmed and 0 = unconfirmed
- 8 and above = high quality
- 5-7 = moderate quality
- 5 and below =low quality
- 85% agreement

Table 1.2 *Modified Critical Appraisal Skills Programme to Evaluate Quality of Studies*

Item Number	Item Description for Experimental Research Designs	Modified Item Description for Observational Research Designs
1	In the study rationale, is there sufficient acknowledgement of essential aspects of theory and pivotal studies?	In the study rationale, is there sufficient acknowledgment of essential aspects of theory and pivotal studies?
2	Did the study address a clearly focused (theory driven) question?	Did the study address a clearly focused (theory driven) question?
3	Was the research design well chosen to address the research question(s)?	Was the research design well chosen to address the research question(s)?
4	Was sample size sufficient or justified using power calculation?	Was sample size sufficient or justified using power calculation?

5*	Was there adequate screening and or testing for significant motor impairment and thus (sufficiently) representative of the proposed population?	Was motor competence of participants identified/screened appropriately?
6*	Did the control group appropriately represent a typically developing population?	Was the overall sample sufficiently representative of the population? (If using a community sampling procedure, for example for DCD, was the proportion of participants identified as at-risk consistent with the prevalence estimates of approximately 5%?)
7	Were the constructs of interests clearly operationalized and measured?	Were the constructs of interests clearly operationalized and measured?
8	Were major confounds adequately controlled?	Were major confounds adequately controlled?
9	Were the statistical methods appropriate and adequately presented?	Were the statistical methods appropriate and adequately presented?
10	Are the major implications of the results clearly discussed?	Are the major implications of the results clearly discussed?

Note - * Consistent with Mancini et al. (2019), studies were given a **score of 1** for using a standardized, clinician administered measures of motor proficiency. Studies that used self-report, parent-report or teach-report measures for motor proficiency were **scored a 0**. Furthermore, studies that did not provide sufficient details were **scored 0** for that item.
